# Feasibility and usability of a non‐immersive virtual reality tele‐cognitive app in cognitive rehabilitation of patients affected by Parkinson's disease

**DOI:** 10.1111/psyg.12880

**Published:** 2022-08-10

**Authors:** Maria Grazia Maggio, Antonina Luca, Concetta D'Agate, Marta Italia, Rocco Salvatore Calabrò, Alessandra Nicoletti

**Affiliations:** ^1^ Department “G.F. Ingrassia”, Section of Neurosciences University of Catania Catania Italy; ^2^ IRCCS Centro Neurolesi “Bonino Pulejo” Messina Italy

**Keywords:** cognitive rehabilitation, feasibility, mobile health, Parkinson's disease, smartphone app, usability

## Abstract

**Background:**

Cognitive impairment is one of the most common non‐motor features of Parkinson's disease (PD). The aim of the present study was to evaluate the feasibility and acceptability/usability of a protocol using a non‐immersive virtual reality tele‐cognitive app, performed remotely in a sample of Italian patients with PD.

**Methods:**

Non‐demented patients with mild PD were included in the study. Patients performed the cognitive rehabilitation in a remote way, at home (three training sessions lasting 20 min/week for 6 weeks) using the NeuroNation app, downloaded for free on the patients' smartphones. The usability and feasibility of the tele‐cognitive rehabilitation program were assessed with the System Usability Scale (SUS) and the Goal Attainment Scaling (GAS).

**Results:**

Sixteen patients (9 men and 7 women; mean age 58.4 ± 8.3 years; mean disease duration 4.6 ± 2.1 years) were included in the study. At the end of the study, the mean SUS was 83.4 ± 11.5. The GAS score recorded at the end of the study (65.6 ± 4.2) was significantly higher than at baseline (38.5 ± 2.4; *P*‐value <0.001).

**Conclusion:**

In our sample, good feasibility and usability were observed for a 6‐week cognitive rehabilitation protocol based on the non‐immersive virtual reality tele‐cognitive app NeuroNation. Our data support the usefulness of cognitive rehabilitation performed in a remote way in PD patients.

## INTRODUCTION

Parkinson's disease (PD) is the second most common neurodegenerative disorder and is characterized by both motor (rest tremor, bradykinesia, rigidity) and non‐motor symptoms (i.e. depression, sleep disorders, cognitive decline).^1^

Cognitive impairment ranging from mild cognitive impairment to dementia, which represents one of the most common and disabling non‐motor symptoms of PD, can be recorded since the early stage of the disease^2^, ^3^, ^4^ and has been associated with a high risk of institutionalization.^5^ Accordingly, the individuation and application of useful rehabilitation/reinforcement cognitive protocols that could represent a strategy for delaying the occurrence or worsening of cognitive deficits are receiving growing interest.^6^ Hence, previous studies have highlighted the beneficial role of cognitive rehabilitation, from traditional paper and pencil methods to more innovative tools, including virtual reality (VR) and PC‐based methods, in PD patients.^7^, ^8^

However, cognitive rehabilitation protocols performed in a hospital setting two to three times a week may not be easy to apply for patients with movement disorders, especially during this SARS‐CoV2 pandemic period, in which most frailty subjects have been advised to avoid hospital visits that are not strictly necessary.^9^, ^10^ In this context, rehabilitation protocols performed remotely could represent a valid alternative to traditional face‐to‐face rehabilitation, to both overcome mobility problems and reduce the cost for the healthcare system.^11^, ^12^ In fact, there are several easy and free digital devices available that allow non‐immersive interactive virtual simulation useful for enhancing cognitive performance in a protected environment.^13^ Moreover, it should be noted that previous studies reported that the clinical efficacy of telerehabilitation could be considered as not inferior to traditional rehabilitation programs. Thus, it seems of interest to evaluate the potential beneficial role for PD patients of telerehabilitation based on free downloaded brain‐training applications (apps) and VR games performed in a remote way. The aim of the present study was to evaluate the feasibility and acceptability/usability of an app combining VR with cognitive telerehabilitation exercises in a sample of Italian patients with PD.

## MATERIAL AND METHODS

### Study population

Non‐demented PD patients diagnosed according to the Movement Disorders Society (MDS) diagnostic criteria,^14^ who attended the ‘Parkinson's Disease and Movement Disorders’ centre of the University Hospital ‘Policlinico‐San Marco’ of Catania between March and November 2021, were enrolled in the study. The inclusion criteria for participation were (i) being aged between 40 and 80 years, (ii) having at least 5 years of schooling, and (iii) having a Hoehn and Yahr (HY) disease stage of <2.5.

The exclusion criteria were (i) the presence of any psychiatric disorders (major depression, psychosis, anxiety disorders), and (ii) a diagnosis of dementia according to the MDS diagnostic criteria.^15^

### Ethics

The study was approved by the University of Catania and was in accordance with the 1964 Helsinki Declaration. Each study participant provided informed consent.

### Clinical and neuropsychological assessment

All of the enrolled patients were evaluated by movement disorders specialists with a standard neurological examination. PD severity was evaluated in an ‘off’ state with the Unified Parkinson Disease Rating Scale—Motor Examination (UPDRS‐ME) and the HY scale. The levodopa equivalent daily dosage (LED) was calculated.

In order to exclude patients suffering from dementia, the Montreal Cognitive Assessment (MoCA), and the Activities of Daily Living and the Instrumental Activities of Daily Living scales were administered.

### VR tele‐cognitive app

A non‐immersive VR tele‐cognitive app called NeuroNation—Brain Training (Synaptikon GmbH, Berlin), available as a free download from the smartphone Play Store, was installed on each patient's smartphone. NeuroNation is an online brain training program offering extensive brain fitness training with a combination of personalized tasks and gamification. The program includes 27 tasks with 250 levels to exercise memory, executive functioning, attention, logical thinking, and thinking speed. The app customizes the tasks based on the user's personal preferences, strengths, and cognitive potential. Audio‐video feedback is provided to encourage performance motivation and self‐assessment during brain training.

### Protocol

#### Baseline visit (face‐to‐face visit)

Once NeuroNation had been downloaded on the patient's smartphone, the level of difficulty was set according to patient characteristics as estimated based on performance (number of errors and time needed) in four time‐limited (1 min) trials aimed at improving various cognitive domains.

#### Subsequent 6 weeks

Patients performed the training remotely at home, following a schedule of three training sessions/week lasting 20 min each for 6 weeks (total number of sessions: 18). The app reminded patients of the training with an alarm at the agreed time (09:00) three times a week (Monday, Wednesday, and Friday). The time for each cognitive domain was standardized among the participants.

At the end of each week, the app proposed a test to ascertain the cognitive level, providing the patient with a performance report. The examiner received the report from the patient every week via text message. In addition, a weekly teleconsultation was carried out to resolve concerns or difficulties.

### Outcome measures

The usability and feasibility of the tele‐cognitive rehabilitation program were assessed with a questionnaire, administered at the end of 6 weeks of treatment, comprising the following scales:

1. System Usability Scale (SUS). This scale provides a reliable ‘quick and dirty’ tool for assessing user acceptance (usability and perception of outcome) regarding hardware, mobile devices, apps, and websites. It consists of 10 items based on the subjective experience of usability. The items are rated on a 5‐point Likert scale ranging from ‘strongly agree’ to ‘strongly disagree.’ Higher scores indicate better usability, while scores <50 indicate ‘difficulties’ in usability^16^, ^17^; scores >60 and <80 are considered ‘good and promising’; and scores of 90 are considered ‘exceptional.’
2. Goal Attainment Scaling (GAS). This scale is used to evaluate the achievement of objectives. The GAS assesses the patient's perception of the goals achieved during the intervention. Each goal is agreed upon with the patient and is evaluated on a 5‐point scale, based on the perceived degree of achievement: −2 = much worse; −1 = a little worse; 0 = expected level; 1 = a little better; 2 = much better.^18^, ^19^ The goal of the present protocol was the ‘improvement of cognitive abilities’.

### Statistical analysis

Statistical analyses were performed using STATA 16.0 software (Stata Statistical Software: Release 16; College Station, TX; StataCorp LLC, 2017). Descriptive statistics were analysed and expressed as mean (standard deviation) for continuous variables as appropriate; frequencies (%) were used for categorical variables. A paired *t*‐test was performed to compare continuous variables, and the chi‐squared test was performed to compare frequencies.

## RESULTS

Sixteen patients (9 men and 7 women; mean age 58.4 ± 8.3 years; mean education 12.9 ± 3.8 years; mean disease duration 4.6 ± 2.1 years) were included, and all completed the study with no dropouts recorded. The mean MoCA score was 26.3 ± 2.4. The mean UPDRS‐ME was 25.1 ± 7.3 and the HY stage was 1.8 ± 0.4. All enrolled patients were treated with levodopa; the LED was 294.7 ± 118.0. No significant differences were found comparing men and women in terms of age (59.2 ± 8.4 and 57.4 ± 8.8 years, respectively; *P* = 0.684) or education (12.1 ± 4.2 and 14.0 ± 3.3 years, respectively; *P* = 0.346).

The mean SUS was 83.4 ± 11.5. Moreover, a statistically significant difference was observed between the GAS scores recorded at baseline (38.5 ± 2.4) and at the end of the study (65.6 ± 4.2; *P* <0.001).

## DISCUSSION

The present pilot study evaluated the feasibility and acceptability/usability of 6 weeks of cognitive telerehabilitation using VR training via smartphone in a sample of non‐demented patients with PD. The novelty of our study is the use of a telerehabilitation system based on digital rehabilitation strategies (mobile health), set and programmed to empower each patient, making him/her autonomous. According to our findings, the NeuroNation app represented a successful protocol in terms of patients’ perception of its usability and achievement of the goal of ‘improvement of cognitive abilities’.

These results lend support to the promise of non‐immersive VR tele‐cognitive apps performed in a remote manner, especially in situations where social distancing is required.

During the COVID‐19 pandemic, in fact, the possibility of supporting patients with telerehabilitation protocols via mobile health, independent of their geographical location, could represent a positive opportunity, considering its accessibility and high motivational power based on the integration of playfulness and gamification.^20^, ^21^, ^22^ Finally, mobile health is flexible, as its use is independent of time and place, and thus adaptable to the daily life of patients.^23^, ^24^ It should be considered that previous studies have reported that patients who consider therapy to be useful and motivating show greater therapeutic adherence and involvement in therapy, especially in distant rehabilitation.^25^, ^26^ Moreover, it has been demonstrated that the use of mobile health can improve functional outcomes, promoting the quality of life of patients, especially in PD.^26^, ^27^ Interestingly, in our sample, the benefit of the telerehabilitation on the quality of life may be supposed from the patients’ frequent requests to use the app on a daily basis.

## STUDY LIMITATION AND CONCLUSION

The study has some limitations. The small sample size may not be sufficient to generalize our findings. Moreover, a possible selection bias due to the inclusion of patients with only mild PD without psychiatric comorbidities cannot be ruled out. However, even though only patients with HY stage <2.5 were enrolled, long disease duration did not represent an exclusion criterion, and not all the enrolled subjects could be considered ‘early PD’. However, the present study was a usability and feasibility pilot study, performed in a selected population of PD patients, preceding the application of the tele‐cognitive rehabilitation protocol in a larger efficacy study.

We believe that further studies using larger samples should be promoted, also evaluating changes in cognitive and emotional functioning.

In conclusion, our pilot study suggests that cognitive telerehabilitation using a smartphone‐based VR app could be an effective way to provide PD patients with home cognitive rehabilitation.

## AUTHOR CONTRIBUTIONS

All authors contributed to the study conception and design. Material preparation, data collection, and analysis were performed by MGM, AL, DAC, IM, RSC, and AN. The first draft of the manuscript was written by MGM and all authors commented on previous versions of the manuscript. All authors read and approved the final manuscript.

## DISCLOSURE

The authors declare no conflict of interests for this article.
